# To report a case of crystalline keratopathy induced by Dieffenbachia plant sap and literature review

**DOI:** 10.1016/j.ajoc.2022.101383

**Published:** 2022-01-31

**Authors:** Elif Bagatur Vurgun, Sıla Cansu Arslan, Semra Akkaya Turhan, Ayşe Ebru Toker

**Affiliations:** Department of Ophthalmology, Marmara University Medical School, İstanbul, Turkey

**Keywords:** Dieffenbachia, Crystalline keratopathy, In vivo corneal confocal microscopy

## Abstract

**Purpose:**

To report a case of crystalline keratopathy induced the Dieffenbachia plant sap.

**Methods:**

Case report and review of the literature.

**Results:**

A 38-year-old woman presented with redness, irritation, and slightly blurred vision in the right eye after the exposure of Dieffenbachia plant sap to her right eye. The patient's eye was irrigated with copious saline on her admission. On ophthalmic examination, her visual acuity was 20/32 OD and 20/20 OS. Anterior segment examination of the right eye revealed mild eyelid edema, grade 2 conjunctival hyperemia, diffuse punctate corneal epithelial erosions, mild stromal edema, and fine refractile needle-like crystals extending from the subepithelial region to mid-stroma. The crystals were visualized with anterior segment photographs and in vivo corneal confocal microscopy (IVCCM) views. Moxifloxacin 0.5% and preservative-free artificial tears were started. Loteprednol etabonate 0.5% was added once the epithelial erosions had healed. The corneal crystals were completely disappeared and the visual acuity of the patient was 20/20 in the third week's visit.

**Conclusions:**

Patients with a history of contact with plant sap should be irrigated with abundant saline immediately to reduce the effect of chemical trauma and thus reduce mechanical damage by inhibiting crystal penetration. IVCCM offers a non-invasive, fast, and reliable diagnosis of Dieffenbachia-related injury, especially in patients with ocular irritation of unknown etiology. Besides, IVCCM is very valuable to differentiate calcium oxalate crystals from other crystalline corneopathies.

## 1 Case report

A splash of sap injured a 38-year-old woman to her right eye while pruning the Dieffenbachia sequine plant. After washing her face with plenty of tap water, she admitted to the emergency department with redness, irritation, and slightly blurred vision in the right eye. Primarily, the patient's eye was irrigated with copious saline. On ophthalmic examination, her visual acuity was 20/32 OD and 20/20 OS. Anterior segment examination of the right eye revealed mild eyelid edema, grade 2 conjunctival hyperemia, diffuse punctate corneal epithelial erosions, mild stromal edema, and fine refractile needle-like crystals extending from the subepithelial region to mid-stroma. The intraocular pressure was normotone; there was no inflammatory reaction within the anterior chamber and vitreous; the fundus was seen as normal. The crystals were visualized with anterior segment photographs and in vivo corneal confocal microscopy (IVCCM) views. Moxifloxacin 0.5% and preservative-free artificial tears four times daily were prescribed to the patient. Two days later, symptoms other than blurring had regressed. On examination, slightly decreased epithelial erosions were seen and previous treatment was continued. On the seventh day of follow-up, the visual acuity was 20/25 OD, and the corneal epithelium was totally healed. Loteprednol etabonate 0.5% ophthalmic suspension was added to treatment. The corneal crystals were gradually reduced and completely disappeared in the third week's visit without sequela. The visual acuity of the patient was 20/20 at the last examination.

## 2 Anterior segment imaging

**Fig. 1:**
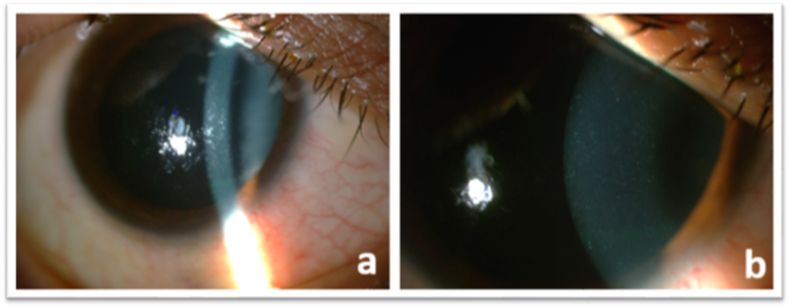
Slit-lamp views showing diffuse needle-like crystals at 16x magnification (a) and at 40x magnification (b).

**Fig. 2:**
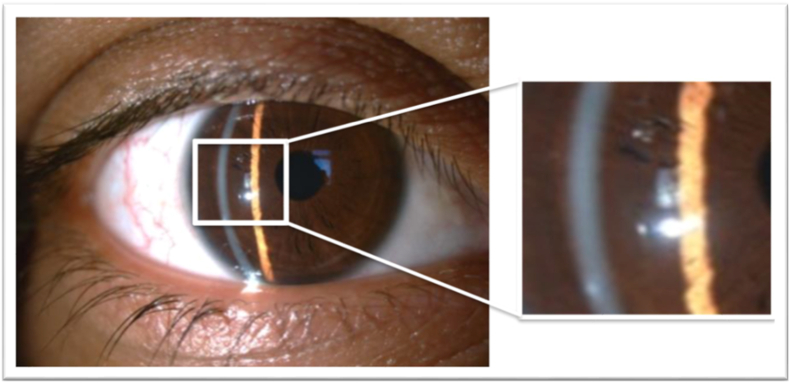
Slit-lamp view showing total clearance of crystals at third week.

The anterior segment photographs were taken at each visit. At first presentation, diffuse fine refractile needle-like crystals extending almost to the posterior stroma were displayed (Fig. 1). At the first week follow-up, the crystals were diminished, and in the third week, complete dissolution was shown (Fig. 2).

## 3 In Vivo Corneal Confocal Microscopy (IVCCM)

The patient's cornea was assessed in vivo by the HRT III in combination with the Rostock Cornea Module-RCM (Heidelberg Engineering). The HRT III/RCM system uses as a laser source a red diode laser (670 nm) and is equipped with a water-immersion lens with a high numerical aperture (636/0.95W; Zeiss). Maintaining the distance between the cornea and the microscope was provided by a disposable contact material (Tomo-Cap). The eye imaged was anesthetized with Proparacaine 0.5% eye drop (Alcaine, Alcon). The contact between the patient's cornea and tomocap was provided by refractive-index matching aqueous gel (Viscotears, Bausch & Lomb).

**Fig. 3:**
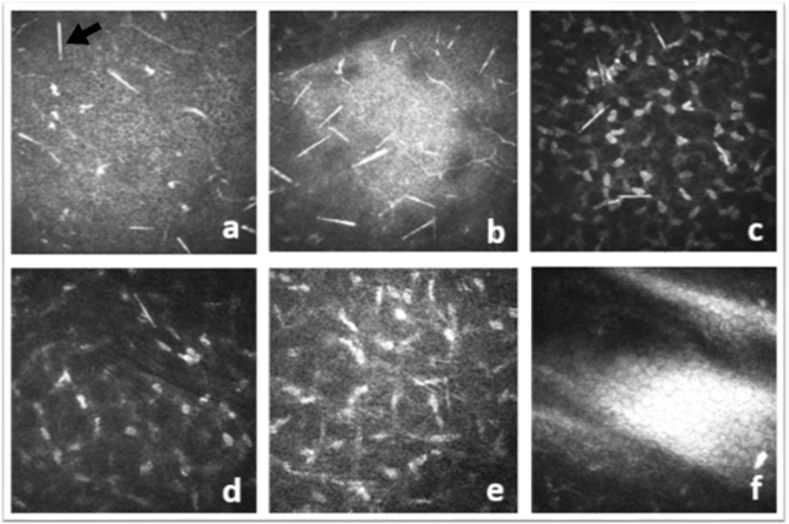
IVCCM views showing highly reflective needle-like structures ‘raphides’ (black arrow) at epithelium (a), bowman layer (b), anterior stroma (c), mid-stroma (d) layers of cornea at first admission. No raphides shown at posterior stroma (e) and endothelium (f) layers.

**Fig. 4:**
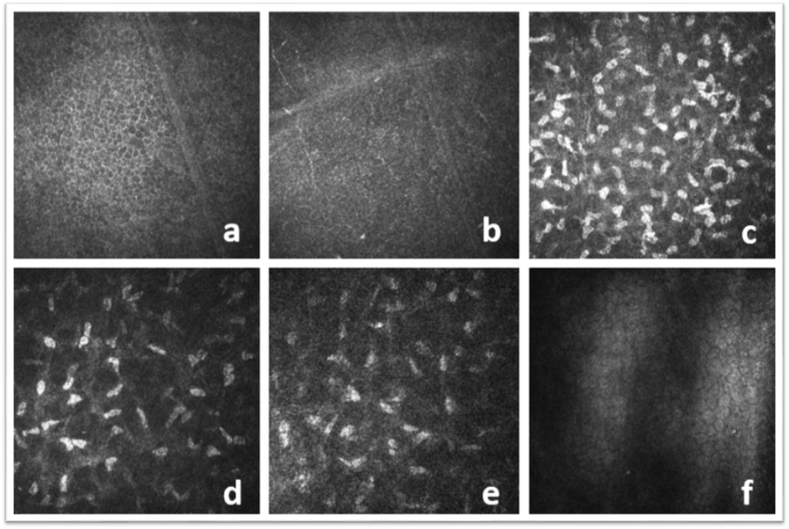
IVCCM views showing no crystalline structure in any corneal layers at third week.

IVCCM examination was carried out at first admission and at the third week when crystals were not clinically visible. The initial analysis revealed highly reflective needle-like structures ranging from 50 to 200 μm in length in all layers from the epithelium to the middle stroma, preserving the corneal structure's integrity (Fig. 3). At the third week, no crystalline structure was observed in any corneal layer (Fig. 4).

## 4 Discussion

Dieffenbachia sequine is a popular household plant belonging to the Araceae family. The plant is known to be poisonous and causes irritation, swelling, and increased secretions when the sap contacts skin or mucous membranes. The plant is called ‘Dumb Cane’ in case of contact with the oral mucosa, the tongue swells, saliva increases, and the person loses the ability to speak for a while.^1^ The toxic mechanism of Dieffenbachia species could not be fully explained by 2020, although it has been researched since 1960.^2^ The most commonly accused toxic agent is calcium oxalate crystals called ‘raphides,’ which are abundant and intensely found in the plant's structure. Many agents, such as a proteolytic enzyme called ‘dumbcain’, cyanogenic glycoside, asparagine, and protoanemoni, have been found to have associated toxigenicity on smooth muscle contraction and soft tissue edema.^3^^,^^4^ There have been several case reports of ocular injuries by Dieffenbachia plant exposure in the literature. Ellis et al. explained the clinical course of ocular injury because of dieffenbachia by an experimental investigation on rabbit corneas.^5^ After the calcium oxalate crystals penetrate the epithelium, they rapidly pass into the anterior stroma. The crystal density decreases towards the posterior part of the cornea. By the eighth day, the crystals significantly reduce, and 75% disappears at the end of the second week. The cornea is slowly cleared from crystals throughout 4–8 weeks without any sequelae. Also, they obtained crystal-free extract by filtering the plant juice, and when they applied it to the cornea, they encountered epithelial necrosis. Fochtman et al. found that a labile protein-like substance caused toxicity in their animal experiment.^6^

In our case, the epithelial erosions reduced on the second day, the corneal epithelium was totally healed by the seventh day, and the visual acuity improved considerably. However, the crystals were still visible at two weeks. Therefore, it could not be stated that only stromal opacity due to crystals play a role in the pathomechanism that mainly affects vision. It is suggested that both chemical trauma caused by proteolytic enzymes and proteinaceous substances that disrupt the epithelial barrier and mechanical trauma caused by calcium oxalate crystals play a role in the pathogenic mechanism. Additionally, the exact mechanism of crystal clearance is unkown. Engulfment and phagocytosis of crystals by dentritic cells is a potential mechanism that is estimated, but no inflammatory cells could be demonstrated by IVCCM.

In the literature, the most common symptoms of the eyes injured with Dieffenbachia species were foreign body sensation, pain, photophobia, and blurred vision. The most common signs were conjunctival injection, corneal epithelial defects, and corneal crystals. Hsueh et al. presented three cases caused by exposure with plants containing milky latex; the case developed due to Dieffenbachia species was more aggressive. The presented visual acuity was lower compared with other patients reported in previous literature. In addition to severe conjunctival swelling and corneal edema, periorbital region involvement was also reported.^7^ Tarsal conjunctival involvement in follicular and papillary reactions was reported in the case presented by Rao et al.^8^ Another patient had presented with iritis in addition to common signs, indicating that the raphides could penetrate full-thickness cornea.^9^ In our patient, absence of clinical findings showing a severe course such as iritis, chemosis, intense eyelid edema was considered due to early irrigation, but also it may be related to the amount of plant water and its expulsive power. Although the general approach was treatment with a combination of antibiotics and steroids, some authors had chosen only antibiotics^10^ or only steroids.^5^^,^^8^^,^^9^^,^^11^ Clinical recovery is seen in 3–7 days, while the duration of corneal crystals disappearance ranges from 3 to 4 weeks^12^, ^13^, ^14^ to 10 weeks.^10^ In the case presented by Vacha et al. the crystals persisted for up to 1 year.^15^ Eventually, the final visual outcomes in reported cases and our case were excellent regardless of the severity and the preferred treatment.

Anciently, detection of calcium oxalate crystals in ocular tissues required pathological and histochemical methods. Zimmerman et al. demonstrated clinically and histopathologically calcium oxalate crystals in the sclerotic lens nuclei of patients with Morgagnian cataracts and in the outer retinal layers of long-term retinal detachments. The formation of crystals was thought to be due to altered biochemical processes, and no crystals were detected apart from these two ocular structures like cornea, sclera, vitreous, uvea or optic nerve.^16^ In reported cases, the diagnosis was based on slit-lamp examination by displaying crystals. As distinct from, Chiou et al. used confocal microscopy for diagnosis, and they performed at first, fourth and eighth weeks. The confocal microscopy provided demonstrative imaging of calcium oxalate crystals (raphides).^11^ Similarly, we also viewed crystals, that are pathognomonic for diagnosis, with confocal microscopy at the first presentation and the third week.

Consequently, patients with a history of contact with plant sap should be irrigated with abundant saline immediately to reduce the effect of chemical trauma and thus reduce mechanical damage by inhibiting crystal penetration. Earlier clearance of crystals may be due to prompt saline irrigation in our patient.

IVCCM offers a non-invasive, fast, and reliable diagnosis of Dieffenbachia-related injury, especially in patients with ocular irritation of unknown etiology. Besides, IVCCM is very valuable to differentiate calcium oxalate crystals from other crystalline corneopathies such as Schnyder dystrophy, Bietti crystalline dystrophy, Richer-Hanhart Syndrome, cystinosis, etc.

## Funding

No funding or grant support

## Authorship

All authors attest that they meet the current ICMJE criteria for Authorship.

## Intellectual property

We confirm that we have given due consideration to the protection of intellectual property associated with this work and that there are no impediments to publication, including the timing of publication, with respect to intellectual property. In so doing we confirm that we have followed the regulations of our institutions concerning intellectual property.

## Research ethics

We further confirm that any aspect of the work covered in this manuscript that has involved human patients has been conducted with the ethical approval of all relevant bodies and that such approvals are acknowledged within the manuscript.

Written consent to publish potentially identifying information, such as details or the case and photographs, was obtained from the patient(s) or their legal guardian(s).

## Contact with the editorial office

This author submitted this manuscript using his/her account in EVISE.

We understand that this Corresponding Author is the sole contact for the Editorial process (including EVISE and direct communications with the office). He/she is responsible for communicating with the other authors about progress, submissions of revisions and final approval of proofs.

We confirm that the email address shown below is accessible by the Corresponding Author, is the address to which Corresponding Author's EVISE account is linked, and has been configured to accept email from the editorial office of American Journal of Ophthalmology Case Reports:

elfbagatur@gmail.com

## Declaration of competing interest

The following authors have no financial disclosures: EBV, SCA, SAT, AET.
